# Ras/MAPK Signaling Modulates VEGFR-3 Expression through Ets-Mediated p300 Recruitment and Histone Acetylation on the *Vegfr3* Gene in Lymphatic Endothelial Cells

**DOI:** 10.1371/journal.pone.0051639

**Published:** 2012-12-17

**Authors:** Taeko Ichise, Nobuaki Yoshida, Hirotake Ichise

**Affiliations:** Laboratory of Developmental Genetics, Center for Experimental Medicine and Systems Biology, The Institute of Medical Science, The University of Tokyo, Minato-ku, Tokyo, Japan; Children’s Hospital Boston & Harvard Medical School, United States of America

## Abstract

Modulation of VEGFR-3 expression is important for altering lymphatic endothelial cell (LEC) characteristics during the lymphangiogenic processes that occur under developmental, physiological, and pathological conditions. However, the mechanisms underlying the modulation of *Vegfr3* gene expression remain largely unknown. Using genetically engineered mice and LECs, we demonstrated previously that Ras signaling is involved not only in VEGFR-3-induced signal transduction but also in *Vegfr3* gene expression. Here, we investigated the roles of the transcription factor Ets and the histone acetyltransferase p300 in LECs in Ras-mediated transcriptional regulation of *Vegfr3*. Ras activates Ets proteins via MAPK-induced phosphorylation. Ets knockdown, similar to Ras knockdown, resulted in a decrease in both *Vegfr3* transcript levels and acetylated histone H3 on the *Vegfr3* gene. *Vegfr3* knockdown results in altered LEC phenotypes, such as aberrant cell proliferation and network formation, and Ets knockdown led to milder but similar phenotypic changes. We identified evolutionarily conserved, non-coding regulatory elements within the *Vegfr3* gene that harbor Ets-binding motifs and have enhancer activities in LECs. Chromatin immunoprecipitation (ChIP) assays revealed that acetylated histone H3 on the regulatory elements of the *Vegfr3* gene was decreased following Ras and Ets knockdown, and that activated Ets proteins, together with p300, were associated with these regulatory elements, consistent with a reduction in *Vegfr3* gene expression in p300-knockdown LECs. Our findings demonstrate a link between Ras signaling and Ets- and p300-mediated transcriptional regulation of *Vegfr3*, and provide a potential mechanism by which VEGFR-3 expression levels may be modulated during lymphangiogenesis.

## Introduction

The lymphatic vasculature is a specialized vascular system that is important for the absorption of macromolecules, tissue fluid homeostasis, and immune cell trafficking. During embryonic development, lymphatic vessels arise from embryonic veins and form a specialized vascular network for lymph flow. In adults, lymph node remodeling, inflammation and tissue repair are accompanied by lymphatic vessel growth, regression and remodeling. Impaired or dysfunctional lymphatic vessel formation causes dysfunction of the lymphatic system, leading to lymphedema, tissue damage and compromised immune responses. It has also been suggested that tumor cell-induced peri- and intratumoral lymphatic vessel formation is involved in tumor spread and development [1].

Genetic studies have demonstrated that VEGFR-3 plays essential roles in endothelial cells (ECs) during developmental lymphangiogenesis [2], [3], [4], [5], although an initial study using knockout mice has shown that VEGFR-3 is essential for cardiovascular development at an earlier stage of embryogenesis [6]. VEGFR-3 tyrosine kinase (TK) activity is required for lymph sac formation by prospero-related homeobox 1 (Prox1)-positive ECs of embryonic veins, vascular endothelial growth factor (VEGF)-C-induced migration of LECs from lymph sacs, and vessel formation by migrated LECs [5], [7]. While VEGFR-3 deficiency leads to embryonic lethality due to impaired cardiovascular development [6], *Vegfr3* haploinsufficiency semi-dominantly induces lymphedema in humans and mice [2], [3]. In humans and mice carrying heterozygous null or heterozygous TK-deficient mutations in the *VEGFR3*/*Vegfr3* gene, the majority of the lymphatic vasculature appears to develop normally. However, the lymphatic capillaries and collecting vessels in peripheral tissues tend to be hypoplastic and cause mild lymphedema, indicating that lymphatic vessel growth and morphogenesis highly depend on the strength of VEGFR-3 signaling. Another line of studies showed that blocking VEGFR-3 signaling *in vivo* with an anti-VEGFR-3 neutralizing antibody inhibits tumor-associated lymphangiogenesis [8] and lymphatic regeneration during wound repair [9] in adults, indicating the involvement of VEGFR-3 in adult lymphangiogenesis. Several studies have also shown that VEGFR-3 expression levels in LECs change during inflammation, and suggest that VEGFR-3 expression levels may modulate LEC responsiveness to VEGFR-3 ligands (VEGF-C and D) and the strength of VEGFR-3 signals, both of which determine LEC behavior [10], [11], [12], [13], [14]. Moreover, dysregulated expression of VEGFR-3 is implicated in lymphangioma formation by LECs [15] and progression of Kaposi’s sarcoma with LEC-like characterisitcs [16], [17]. Collectively, these data confirm that *VEGFR3* gene expression levels are influential in developmental, physiological and pathological lymphangiogenesis.

The promoter region of the *Vegfr3* gene was initially identified by reporter assays in cells and transgenic mouse embryos [18]. Subsequent studies demonstrated that overexpression of CBF-1/suppressor of hairless/Lag1 (CSL)-activating mutant Notch [19], NF-kB family proteins (p50 and p65) [11] and Prox1 [11], [13], [20], [21], [22], [23] upregulate *VEGFR3* promoter-driven reporter expression and/or endogenous *VEGFR3* gene expression in blood ECs (BECs) [11], [13], [19], [20], [21], [22], [23] and 293T cells [23]. Moreover, it has been shown that knockdown of NF-kB p50/p65 [11] and Prox1 [22], [24] results in decreased VEGFR-3 expression levels in LECs, and that endogenous NF-kB p50/p65 [11], overexpressed CSL-activating mutant Notch [19], Prox1 [13], [23] and E26 avian leukemia oncogene (Ets) 2 [13] bind the endogenous *VEGFR3* promoter region, suggesting that those transcription factors might transactivate *VEGFR3* gene expression via the promoter. On the other hand, a regulatory region other than the promoter has also been postulated to be important for *Vegfr3* gene expression. Chen et al. showed that mice lacking the transcription factor T-box 1 (Tbx1) in an EC lineage exhibited abnormal intestinal lymphatic vessel development, and identified a Tbx1-responsive enhancer element in an intronic region of the *Vegfr3* gene. These findings suggest that Tbx1-mediated transcriptional regulation of the *Vegfr3* gene may be important for the growth and maintenance of lymphatic vessels [25]. Nevertheless, the precise mechanism of transcriptional regulation of *Vegfr3* expression remains largely unknown.

Previously, we found that Ras knockout mice and transgenic mice overexpressing H-Ras in an endothelial cell lineage exhibit lymphatic vessel hypoplasia and hyperplasia, respectively [26]. Using immortalized mouse LECs *in vitro*, we demonstrated that Ras expression levels positively correlate with the activity levels of Ras and its downstream targets, MAPKs, in response to stimuli by serum and VEGFs [26]. Additionally, we found that *Vegfr3* gene expression is up-regulated by active Ras, suggesting that Ras plays an important role not only in VEGFR-3 downstream signaling, but also in modulation of *Vegfr3* gene expression in LECs [26]. However, the underlying mechanism by which Ras signaling modulates *Vegfr3* gene expression remains elusive.

The Ets transcription factors, Ets1 and Ets2, are MAPK substrates and regulate the transcription of genes that harbor GGAA/T motif-containing regulatory regions [27]. These proteins are known to be evolutionarily conserved, nuclear downstream effectors of the Ras/MAPK pathway [28], [29], [30], [31]. In addition, another line of studies suggests that Ets proteins may be involved in the transcriptional regulation of EC marker genes [32], [33]. In the present study, we demonstrate that the transcription factors Ets1 and Ets2 act as downstream effectors of the Ras/MAPK pathway and participate in *Vegfr3* gene expression in LECs. Ets proteins, upon activation by Ras/MAPK signaling, bind to 5′ upstream- and intronic regions of the *Vegfr3* gene whose nucleotide sequences are highly conserved among species. Ets proteins recruit the histone acetyltransferase p300 to the *Vegfr3* gene, leading to histone acetylation and transcriptional activation of the *Vegfr3* promoter.

## Results

### Ets1 and Ets2 Act as Downstream Effectors of Ras/MAPK Signaling and Participate in *Vegfr3* Gene Expression in LECs

**Figure 1:**
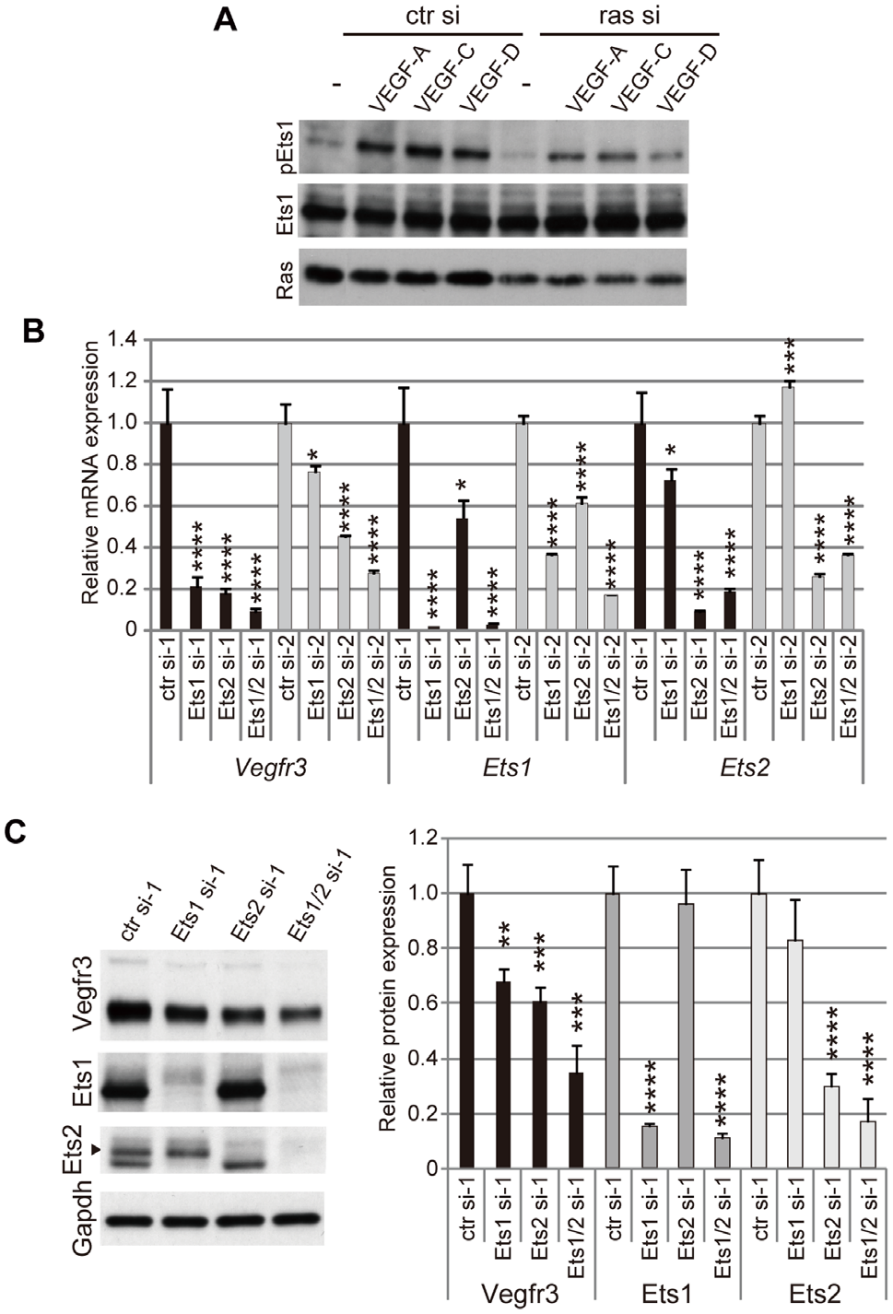
Ets proteins are involved in VEGFR-3 expression and VEGFR signaling. A. Western blots of phosphorylated Ets1 (pEts1), Ets1 and total Ras proteins in immortalized mouse LECs (mLECs) transfected with control siRNA or mixed siRNAs for *ras* genes (*Hras*, *Nras* and *Kras*) in the presence or absence of VEGF-A, VEGF-C or VEGF-D. B. Real-time RT-PCR assay for mRNAs in mLECs transfected with control, Ets1 and Ets2 siRNAs. si-1 and -2 represent two individual siRNAs. Ets1/2 si represents transfection with mixed siRNAs for Ets1 and Ets2. Error bars represent the S.D.; n = 3. *p<0.05, ***p<0.005, ****p<0.001 (vs. mLECs transfected with control siRNA; see Table S2 and Table S3). C. Protein expression in mLECs transfected with control, Ets1 and Ets2 siRNAs. Left panel, western blots; right panel, quantitative analysis of western blots. Error bars represent the S.D.; n = 3. **p<0.01, ***p<0.005, ****p<0.001 (vs. mLECs transfected with control siRNA).

To elucidate whether Ets proteins are involved in control of VEGFR-3 expression, we examined whether Ets proteins become phosphorylated through Ras/MAPK signaling using immortalized, mouse mesenteric LECs (mLECs). Previously, we showed that VEGF-A, C and D stimulate activation of Ras and MAPKs in mLECs, and that VEGF-induced activation of Ras/MAPK signaling is suppressed by Ras knockdown [26]. In similar experiments, Ets1 was phosphorylated in response to VEGF-A, C and D, and Ras knockdown abrogated VEGF-dependent phosphorylation of Ets1 compared to Ets1 in starved mLECs with mock treatment (Fig. 1A). Because growth factors other than VEGFs can also induce Ets1 phosphorylation in mLECs (data not shown), we cannot definitively state that this signaling cascade is responsible for Ets1 phosphorylation; however, these results strongly suggest that Ras/MAPK signaling-dependent phosphorylation of Ets proteins may mediate transcriptional activation of the *Vegfr3* gene. We next performed knockdown experiments to elucidate whether Ets proteins play a role in *Vegfr3* gene expression. We knocked down both Ets1 and Ets2 expression, because these proteins are highly homologous and appear to be functionally indistinguishable [27]. Following knockdown of either Ets1 or Ets2, *Vegfr3* mRNA expression was decreased as demonstrated by real-time RT-PCR (Fig. 1B). This decrease in *Vegfr3* mRNA resulted in a decrease in VEGFR-3 protein expression (Fig. 1C). In addition, Ets1/Ets2 double knockdown further decreased VEGFR-3 expression (Fig. 1B and 1C), suggesting that Ets1 and Ets2 may function redundantly and cooperatively in the transcriptional regulation of *Vegfr3*. Ets family proteins have been reported to activate transcription from the promoters of many endothelial cell-specific genes including *Vegfr2*, *Pecam1* and *VEcadherin* [32], [33]. Therefore, we examined changes in the mRNA expression levels of those genes. Ets1/2 knockdown by either the si-1 or si-2 series of siRNAs resulted in a slight but statistically significant decrease in *Vegfr2* and *VEcadherin* expression (Fig. S1A); however, neither Ets1 nor Ets2 knockdown reduced *VEcadherin* mRNA levels significantly, and controversial changes were observed in *Vegfr2* and *Pecam1* mRNA expression in mLECs transfected with the si-1 and si-2 siRNAs (Fig. S1A). Although different siRNAs to the same gene with comparable knockdown efficacies should induce similar changes in gene expression profiles, variability in gene expression profiles induced by different siRNAs to the same gene is thought to be caused by siRNA-specific off-target effects. Thus, these results suggest that the maintenance of *Vegfr2*, *Pecam1* and *VEcadherin* gene expression levels might not be dependent on the presence of Ets1 or Ets2 in mLECs. Ets family proteins other than Ets1 and Ets2 may be able to compensate for the lack of Ets1/2 expression. In contrast, both of the Ets siRNAs reduced *Vegfr3* mRNA expression. We then examined the mRNA expression levels of three housekeeping genes, *Gapdh*, *Hprt*, and *Tbp*, and normalized *Vegfr3* mRNA levels to these genes. As shown in Fig. S1B, *Vegfr3* mRNA levels normalized to the mRNA levels of these genes show a reduction in expression comparable to that observed when normalized to 18S rRNA (Fig. 1B). Collectively, these results suggest that the transcription of *Vegfr3* may be highly dependent on the strength of Ras/MAPK/Ets signaling and the levels of Ets1 and Ets2 in LECs.

### Ets1 and Ets2 are Involved in LEC Proliferation and Network Formation *in vitro*

**Figure 2:**
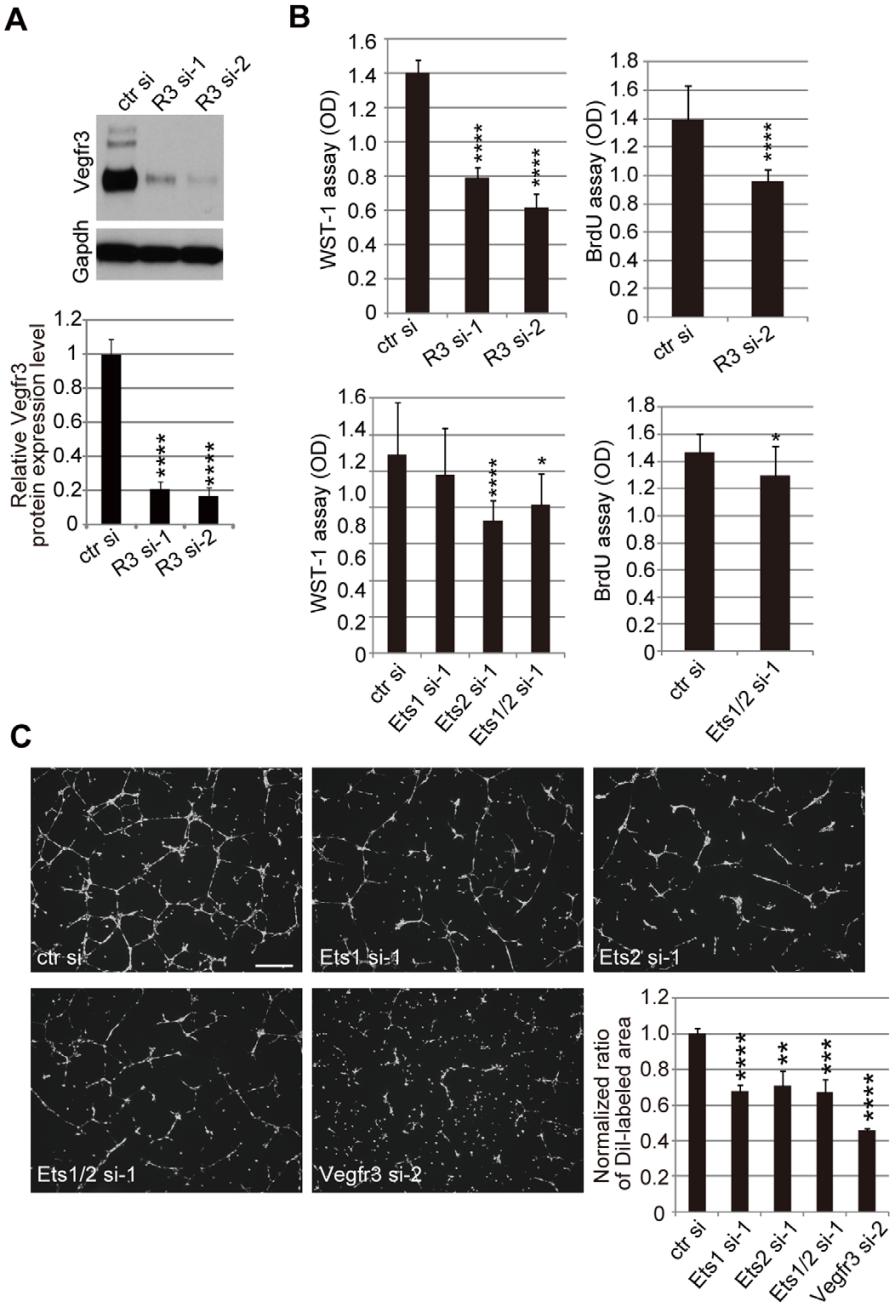
Impact of Ets and VEGFR-3 knockdown on mLECs *in vitro*. A. VEGFR-3 and Gapdh protein expression in Vegfr3-knockdown LECs. Upper panel, western blots; lower panel, quantitative analysis of western blots. Error bars represent the S.D.; n = 3. ****p<0.001 (vs. mLECs transfected with control siRNA). B. WST-1 assays using Vegfr3-knockdown mLECs (upper left panel) and Ets-knockdown mLECs (lower left panel), and BrdU assays using Vegfr3-knockdown mLECs (upper right panel) and Ets-knockdown mLECs (lower right panel). Error bars represent the S.D.; n = 12. *p<0.05, ****p<0.001 (vs. mLECs transfected with control siRNA in each assay). C. DiI-stained cellular networks of mLECs transfected with control, Ets1, Ets2, and Vegfr3 siRNAs on Matrigel. Scale bar = 500 µm. DiI-labeled areas were quantified and the mean area of DiI-labeled wild-type mLECs was normalized to 1. Error bars represent the S.D.; n = 3. **p<0.01, ***p<0.005, ****p<0.001 (vs. mLECs transfected with control siRNA).

In order to determine whether Ets-mediated changes in VEGFR-3 expression levels have an effect on LEC phenotypes, we examined cell proliferation and network formation on Matrigel by Ets- and VEGFR-3-knockdown mLECs. Reduction of VEGFR-3 resulted in reduced proliferation of mLECs (Fig. 2A and 2B) and impaired network formation (Fig. 2C), as expected. Although Ets2 knockdown, but not Ets1 knockdown, reduced cell proliferation (Fig. 2B), both Ets1 and Ets2 knockdown resulted in mild but significant impairment of network formation (Fig. 2C). These results indicate that endogenous Ets, as well as VEGFR-3, functions in mLECs in terms of cellular proliferation and morphogenesis. Although we cannot exclude the possibility that Ets may function in mLECs by regulating transcription of genes other than *Vegfr3*, the results imply that Ets-mediated maintenance of VEGFR-3 expression levels may contribute to VEGFR-3-dependent mechanisms responsible for mLEC phenotypes.

### ETS1-knockdown in Human LECs Leads to a Decrease in *VEGFR3* Gene Expression, Reduced Proliferation and Impaired Network Formation

**Figure 3:**
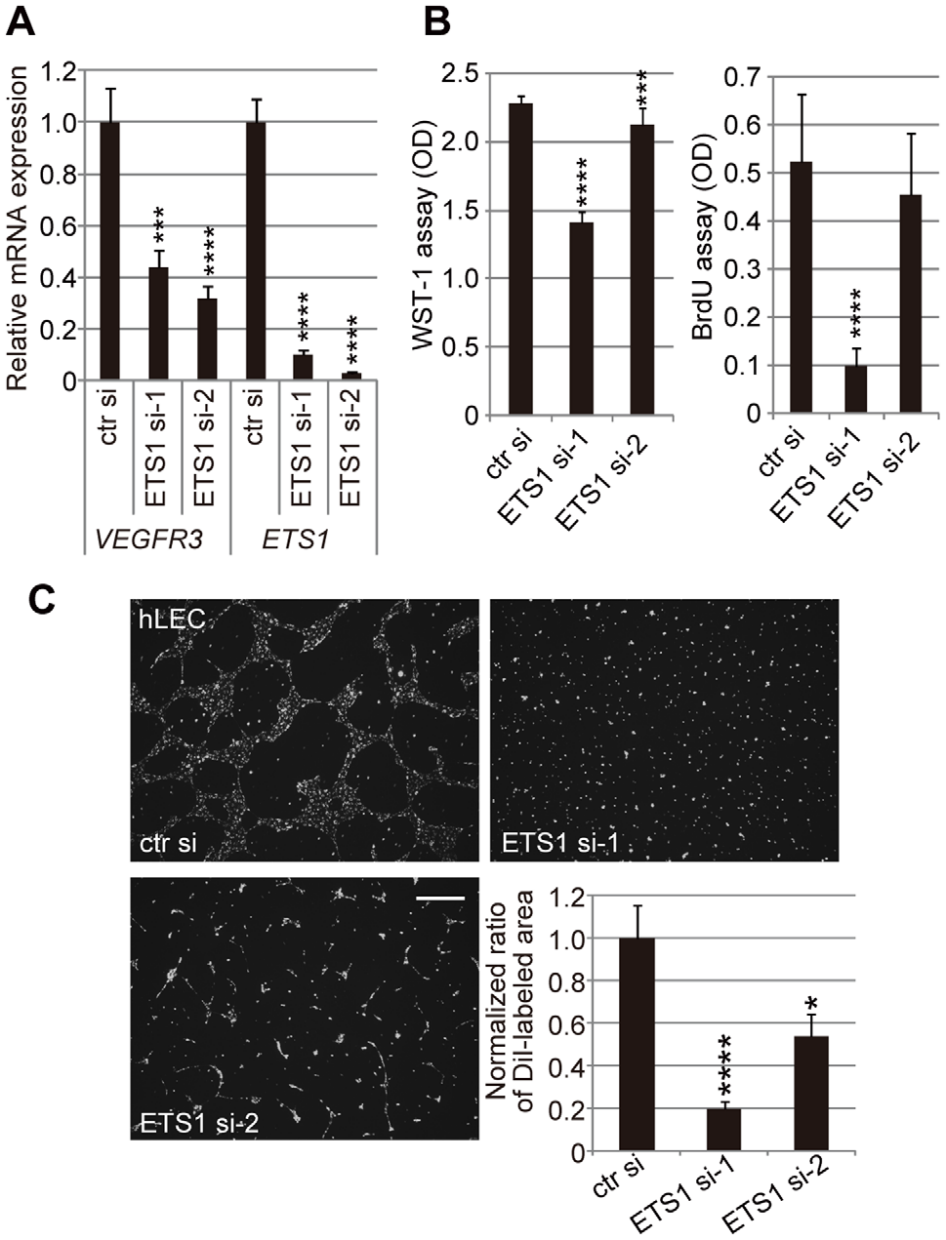
Human ETS1 is involved in *VEGFR3* gene expression, cell proliferation and network formation by human LECs. A. Real-time RT-PCR assay for *VEGFR3* mRNA in primary human LECs (hLECs) transfected with control and ETS1 siRNAs. si-1 and -2 represent two individual siRNAs. Error bars represent the S.D.; n = 3. ***p<0.005, ****p<0.001 (vs. hLECs transfected with control siRNA; see Table S6). B. WST-1 assays and BrdU assays using ETS1-knockdown hLECs. Error bars represent the S.D.; n = 12. ***p<0.005, ****p<0.001 (vs. hLECs transfected with control siRNA). C. DiI-stained cellular networks of hLECs transfected with control and ETS1 siRNAs on Matrigel. Scale bar = 500 µm. DiI-labeled areas were quantified and the mean area of DiI-labeled wild-type hLECs was normalized to 1. Error bars represent the S.D.; n = 3. *p<0.05, ****p<0.001 (vs. hLECs transfected with control siRNA).

To rule out the possibility that Ets-mediated *Vegfr3* gene expression might be a mouse-specific or immortalized cell-specific phenomenon, we performed knockdown experiments in human primary LECs. We found that while ETS1 knockdown reduced *VEGFR3* gene expression (Fig. 3A), cell proliferation (Fig. 3B), and network formation (Fig. 3C), ETS2 knockdown did not result in the reduced expression of *VEGFR3* (data not shown). Despite this discrepancy between the roles of Ets2/ETS2 in mouse and human LECs, these results strongly suggest that Ets/ETS proteins modulate *Vegfr3*/*VEGFR3* gene expression, cellular proliferation, and morphogenesis in both mouse and human LECs.

### Ets1 and Ets2 Direct Transcription of the *Vegfr3* Promoter in a Ras Signaling-dependent Manner

**Figure 4:**
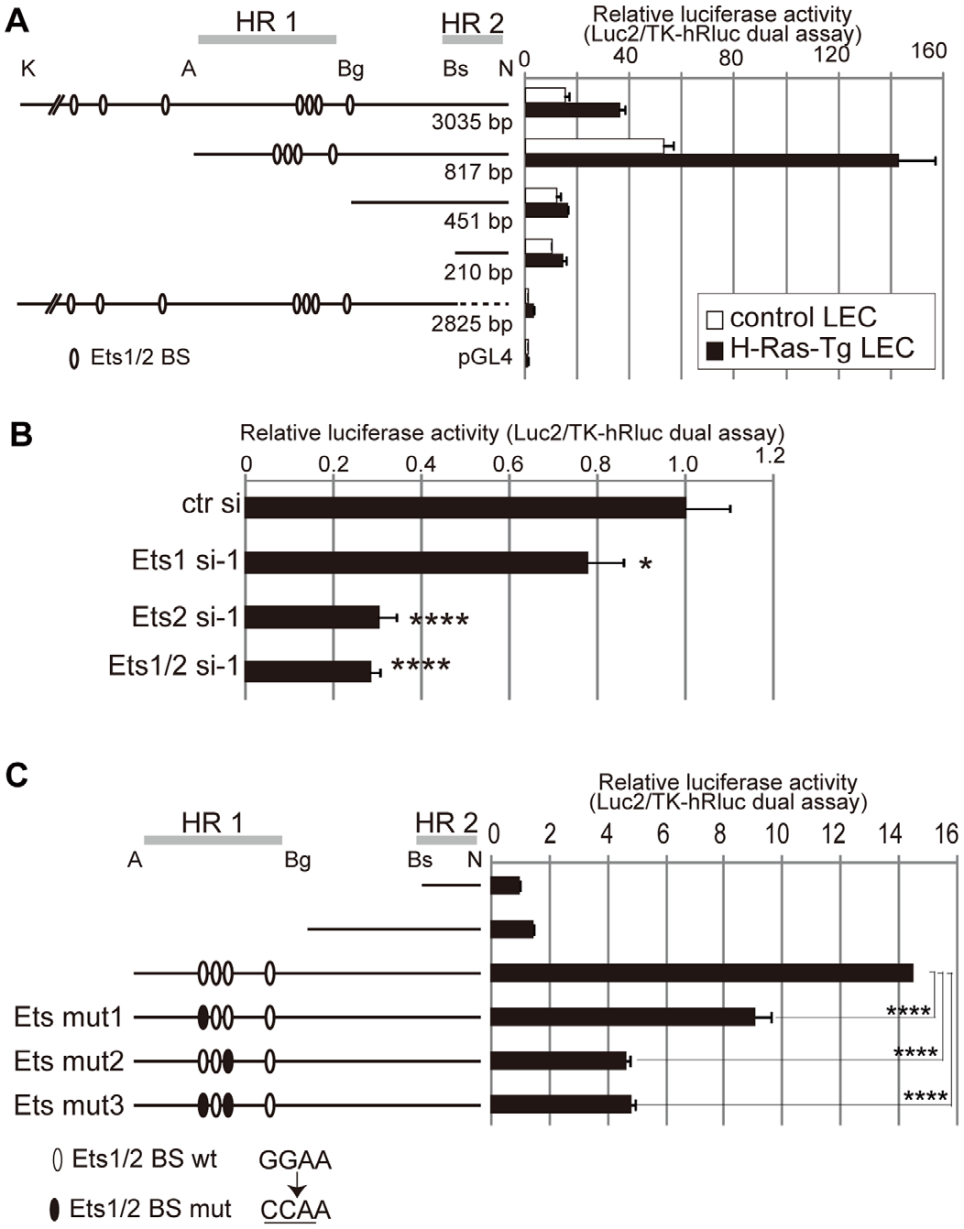
The 5′ regulatory region of the *Vegfr3* gene drives luciferase expression in an Ets-dependent manner. A. Luciferase assay using the pGL4-luc2 vector harboring variable lengths of the 5′ untranscribed and UTR regions of the mouse *Vegfr3* gene in control and H-Ras-overexpressing mLECs. K, Kpn I; A, Avr II; Bg, Bgl II; Bs, BsiWI; N, Not I. A dashed line indicates the deletion between the BsiWI and Not I sites. Open ovals show putative Ets-binding sites predicted by the Transcription Element Search System (TESS; http://www.cbil.upenn.edu/tess). The previously reported homologous regions HR1 and HR2 [18] are also shown. Relative luciferase activities (luc2 vs. TK-hRluc vector-produced hRluc) are shown. Error bars represent the S.D.; n = 3. B. Luciferase assay using the pGL4-Luc2 vector harboring the Avr II-Not I fragment in control and Ets knockdown mLECs. Error bars represent the S.D.; n = 3. *p<0.05, ****p<0.001 (vs. mLECs transfected with control siRNA). C. Luciferase assay using the pGL4-Luc2 vector harboring the Avr II-Not I fragment with or without mutated Ets-putative binding sites (GGAA-to-CCAA mutation). Reporter vectors harboring the Bs-N fragment or Bg-N fragment were also assayed for comparison. Error bars represent the S.D.; n = 3. ****p<0.001 (vs. the wild-type Avr II-Not I fragment).

To elucidate how Ets proteins regulate *Vegfr3* gene expression, we performed luciferase assays using the promoter region of the mouse *Vegfr3* gene. It was shown previously that the 5′ untranscribed regions of the mouse and human *Vegfr3* genes contain two highly conserved regions [18]. One of these regions (previously named HR1) was reported to be a regulatory region that contains Ets-binding consensus sites between the Avr II and Bgl II sites, and the other (HR2) was identified as a core promoter region included in the fragment between BsiWI and Not I sites (Fig. 4A) [18]. To identify a Ras signaling-responsive element in the 5′ untranscribed region of the mouse *Vegfr3* gene, the luciferase activity of a *Vegfr3*-Luc construct series was compared in control and H-Ras-overexpressing mLECs. Because Ras/MAPK signaling and increased VEGFR-3 expression has been observed in H-Ras-overexpressing mLECs [26], luciferase activity was expected to be higher in H-Ras-overexpressing mLECs compared to control mLECs. Not only did the Avr II-Bgl II fragment (HR1) in the promoter region increase luciferase expression, as demonstrated previously, but this region activated luciferase expression more strongly in H-Ras-overexpressing mLECs than control mLECs (Fig. 4A). To determine the role of Ets in the enhancer activity of the region, we assayed the luciferase activity of the HR1-containing reporter vector in Ets knockdown mLECs and found that Ets knockdown downregulated luciferase activity (Fig. 4B). We next performed luciferase assays using constructs harboring mutated Ets-binding sites in the HR1 region (Ets mut1, mut2 and mut3) and found that Ets-binding site mutations, especially Ets mut2, reduced transcription (Fig. 4C). These results suggested that Ets binding to the HR1 region might be important for *Vegfr3* gene expression. However, in newly-generated knock-in mice of *Vegfr3*-EGFP harboring Ets-binding site mutations, neither EGFP expression levels nor expression pattern were affected significantly compared with the wild-type *Vegfr3*-EGFP knock-in mice described previously [26] (Ichise, T., unpublished observation). To explain the discrepancy between the results obtained from luciferase assays and assays using reporter mice, we speculated that intronic regions other than the 5′ untranscribed region might also participate in Ets-mediated regulation of endogenous *Vegfr3* gene expression, redundantly and/or cooperatively with the 5′ untranscribed region.

### Evolutionarily Conserved Regions in the *Vegfr3* Gene Harbor Ets-binding Motifs with Enhancer Activities

**Figure 5:**
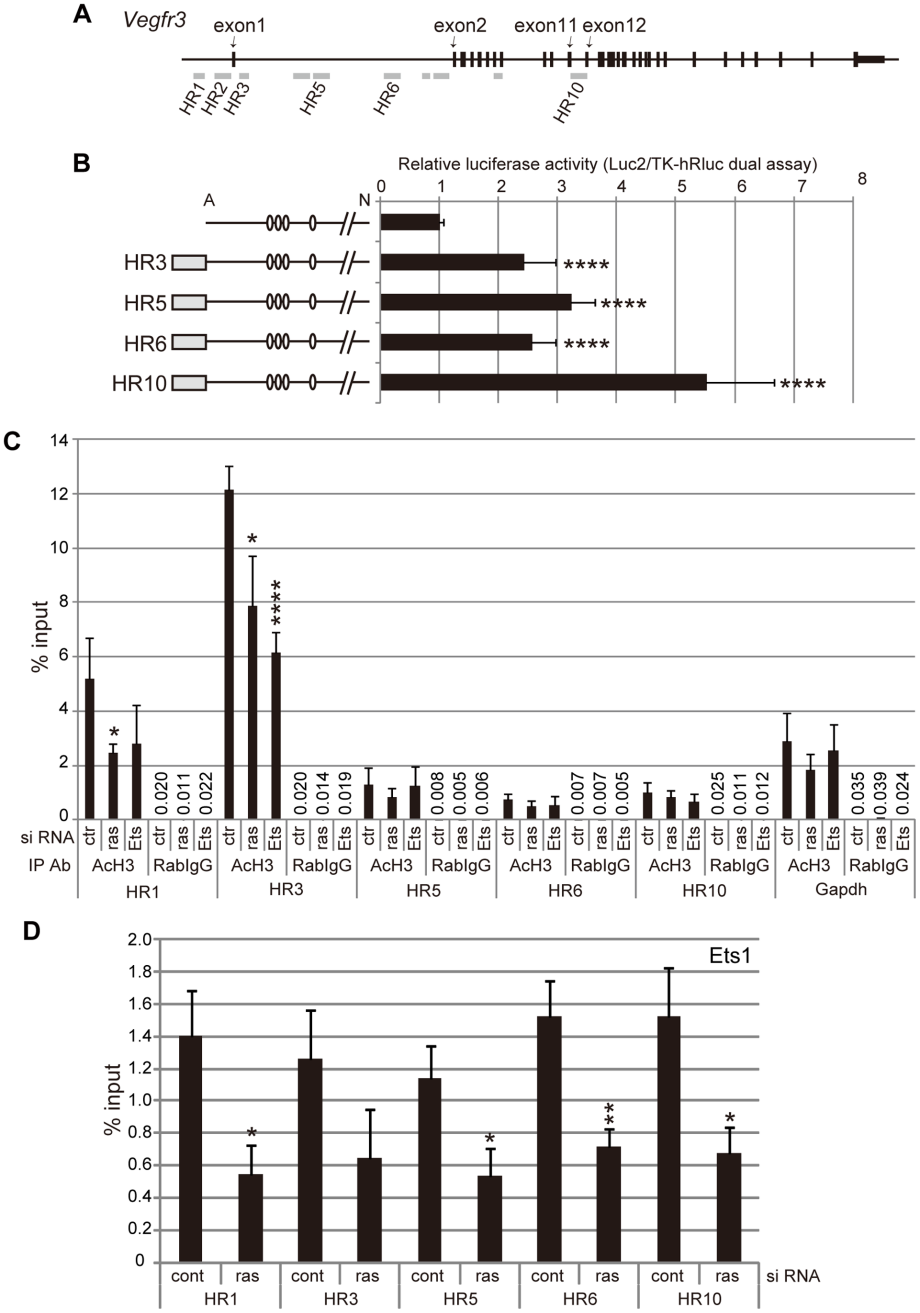
Evolutionarily conserved regions in the *Vegfr3* gene act as transcription enhancers in Ras dose-dependent and Ets-dependent manners. A. Schematic representation of evolutionarily conserved regions in the mouse *Vegfr3* gene. HR1 and HR2 were identified and described in a previous study [18]. For details, see Fig. S2 and Fig. S3. B. Luciferase assay using the pGL4-Luc2 vector harboring the Avr II-Not I fragment (described in Fig. 4) and fragments derived from HR 3, 5, 6 or 10 in mLECs. The HR fragments used in this study are described in Fig. S3. Error bars represent the S.D.; n = 3. ****p<0.001 (vs. the HR3-10 fragment-free vector). C. ChIP assay showing H3 histone acetylation on the *Vegfr3* gene in control, Ras- and Ets-knockdown mLECs. Error bars represent the S.D.; n = 3. *p<0.05, ****p<0.001 (vs. mLECs transfected with control siRNA). Numerical values of the means are shown as % input for rabbit IgG-immunoprecipitated, sonicated chromatin. Results of PCR for *Gapdh* are shown for comparison. D. ChIP assay showing Ets1 binding to the evolutionarily conserved regions of the *Vegfr3* gene in control and Ras-knockdown mLECs. Error bars represent the S.D.; n = 3. *p<0.05, **p<0.01 (vs. mLECs transfected with control siRNA).

To identify the intronic regions potentially responsible for Ets-mediated transcriptional regulation, we explored evolutionarily conserved regions in introns of the *Vegfr3* gene. Using the UCSC genome browser, the DNA sequences of mammalian or vertebrate orthologs of the *Vegfr3* gene were compared. All of the conserved sequences that were identified contained GGAA/T motifs (Fig. 5A, Fig. S2 and Fig. S3). Based on the search results, 10 genomic regions of the *Vegfr3* gene were selected and designated HR1 to HR10, including the regions formerly-defined as HR1 and HR2. The intronic region between exon 11 and 12, identified as HR10 in this study, contains a FOX:ETS motif [34] and a Tbx1-binding site that has been reported to be important for Tbx1-dependent enhancer activity in ECs [25]. To elucidate whether the evolutionarily conserved regions have enhancer activities, luciferase assays were performed using reporter constructs carrying Ets-binding motif-containing fragments within the intronic HR regions. Transcription in these reporter constructs was driven by the HR1- and HR2-containing *Vegfr3* promoter region (Fig. 5B). All of the fragments examined (four out of seven intronic HRs) up-regulated reporter expression in mLECs (Fig. 5B), suggesting that these evolutionarily conserved intronic regions may be involved in the regulation of *Vegfr3* gene expression.

### Ras/MAPK/Ets Signaling Regulates the Transcriptional Activity of the *Vegfr3* Gene

To determine whether Ras/MAPK/Ets-dependent changes in *Vegfr3* transcripts depend on transcriptional regulation, we examined H3 histone acetylation events on the *Vegfr3* gene in control-, Ras- and Ets-knockdown mLECs. H3 histone acetylation events represent transcriptionally active regions, especially those surrounding the transcription start sites of transcribed genes [35]. ChIP assay using anti-acetylated H3 histones revealed that the HR1 and HR3 regions contained larger amounts of acetylated H3 histone than regions HR5, HR6 and HR10, which are farther from the core promoter (Fig. 5C). Moreover, Ras and Ets knockdown led to a decrease in histone H3 acetylation in the HR1 and HR3 regions of the *Vegfr3* gene, whereas acetylated histone levels on the *Gapdh* gene were not affected by Ras or Ets knockdown (Fig. 5C). These results suggest that Ras/MAPK/Ets signaling regulate the transcriptional activity of the *Vegfr3* gene.

To further elucidate whether Ras-dependent transcriptional regulation of the *Vegfr3* gene involves Ets binding, we performed ChIP assay using anti-Ets1. Ets bound to all evolutionarily conserved regions that were examined, and the amount of Ets1 binding to those regions was decreased by Ras knockdown (Fig. 5D). These results are consistent with the finding that these regions contain Ets-binding consensus sequences (Fig. S3) and previous reports showing that Ras/MAPK-induced phosphorylation enhances the transcriptional activity of Ets [28], [29], [30], [31]. Thus, these results suggest that Ras signaling-induced Ets binding to *Vegfr3* enhancer regions is involved in *Vegfr3* gene expression. Although we could not examine Ets2 binding due to the lack of an anti-Ets2 antibody suitable for ChIP, our findings strongly suggest that Ets2 mediates *Vegfr3* gene expression in mLECs (Fig. 1, Fig. 2 and Fig. 4). It is probable that Ets2 binds to the HR regions in a manner similar to Ets1.

### Ets Proteins Bind to Intact Chromatin of the *Vegfr3* Gene and Recruit the Histone Acetyltransferase p300

**Figure 6:**
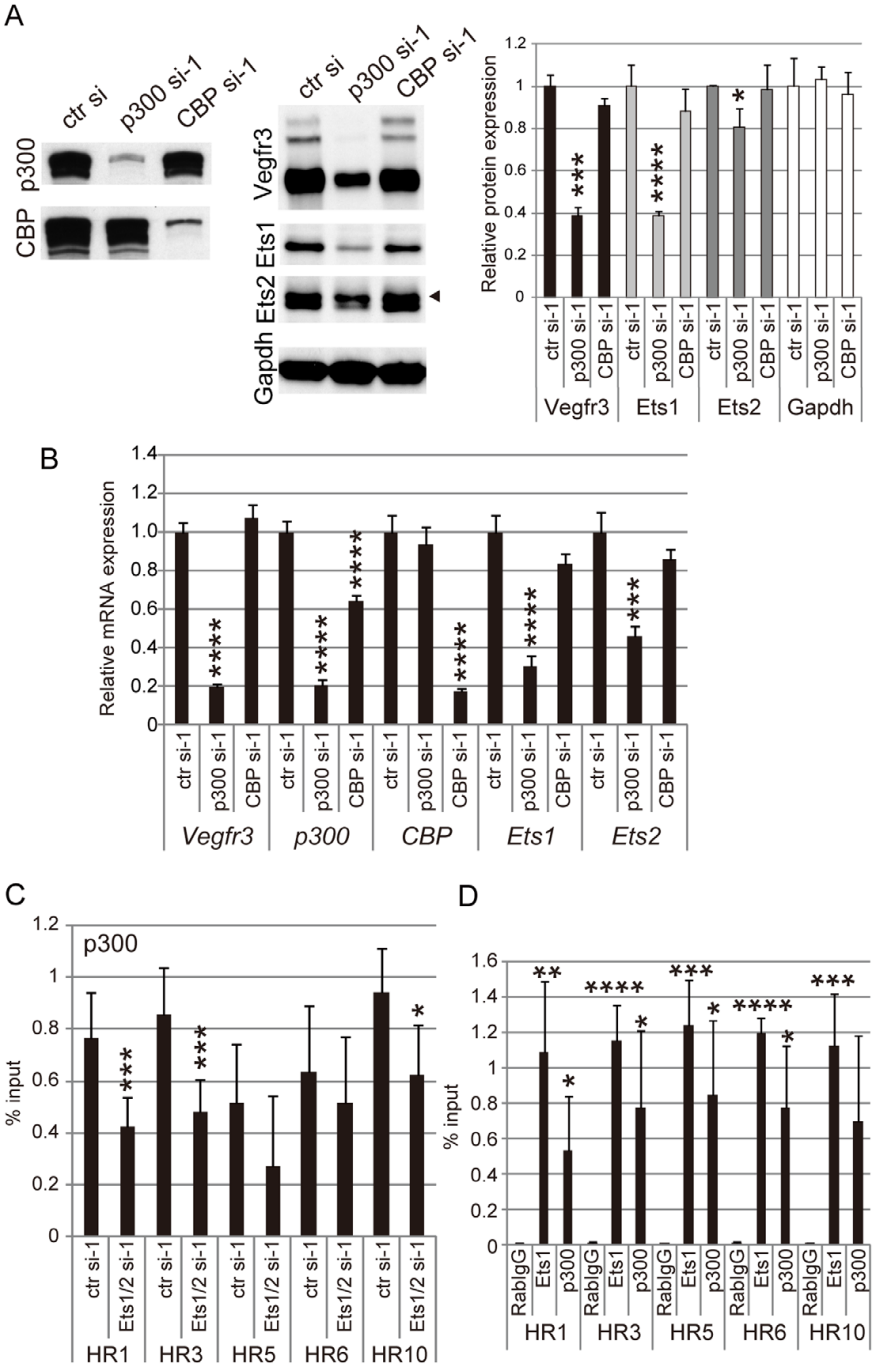
Ets mediates histone acetyltransferase p300 recruitment to non-coding regions of the *Vegfr3* gene. A. Protein expression in mLECs transfected with control, p300 and CBP siRNAs. The left and middle panels show western blots for p300, CBP, VEGFR-3, Ets1, Ets2 and Gapdh. The right panel shows quantitative analysis of western blots. Error bars represent the S.D.; n = 3. *p<0.05, ***p<0.005, ****p<0.001 (vs. mLECs transfected with control siRNA). B. Real-time RT-PCR assay for *Vegfr3*, *p300*, *CBP, Ets1* and *Ets2* mRNAs in mLECs transfected with control, p300 and CBP siRNAs. Error bars represent the S.D.; n = 3. ***p<0.005, ****p<0.001 (vs. mLECs transfected with control siRNA; see Table S7). C. ChIP assay showing p300 association with the evolutionarily conserved regions of the *Vegfr3* gene in control and Ets1-knockdown mLECs. Error bars represent the S.D.; n = 3. *p<0.05, ***p<0.005 (vs. mLECs transfected with control siRNA). D. ChIP assay showing Ets1- and p300 association with the evolutionarily conserved regions of the *Vegfr3* gene in the small intestine of newborn mice. Error bars represent the S.D.; n = 3. *p<0.05, **p<0.01, ***p<0.005, ****p<0.001 (vs. rabbit IgG).

Previous studies suggest that transcriptional activation by Ets proteins involves recruitment of histone acetyltransferases (HATs) onto genes. MAPK-induced phosphorylation of Ets leads to Ets-HAT interaction, which enhances the transcriptional activity of Ets [36], [37]. Two types of HATs, p300 (also known as Ep300) and CREB binding protein (CBP; also known as Crebbp), are known to be cofactors of Ets for transcriptional activation. These proteins do not bind directly to chromatin, but instead bind to a variety of DNA-binding proteins, including Ets proteins [35]. To determine whether p300 and CBP participate in Ets-mediated *Vegfr3* gene expression, we knocked-down p300 and CBP in mLECs and examined the effects on VEGFR-3 expression. As shown in Fig. 6A, Fig. 6B and Fig. S4, VEGFR3 expression was downregulated by p300 knockdown but not by CBP knockdown, suggesting that the transcriptional changes observed for *Vegfr3* result from gene-specific regulation by Ets1/2 and p300. However, we also hypothesized that p300 might modulate *Vegfr3* gene expression by regulating Ets expression rather than being associated with Ets on the *Vegfr3* gene, since p300 knockdown also decreased Ets1 and Ets2 expression (Fig. 6A, Fig. 6B and Fig. S4). To confirm whether the role of p300 in regulating *Vegfr3* gene expression is based on Ets-assisted recruitment to *Vegfr3*, ChIP assay was performed to examine the association of p300 with the *Vegfr3* gene. The results showed that p300 was associated with the promoter and the intronic regions of the *Vegfr3* gene bound by Ets, as described above, and that Ets knockdown resulted in the reduced association of p300 with the HR1, HR3 and HR10 regions (Fig. 6C). Additionally, Ets1/p300 recruitment onto the intact chromatin of the *Vegfr3* gene was also revealed by ChIP assay on LEC-rich, mouse intestine tissue samples (Fig. 6D). These results suggest that p300 may be associated with these regulatory regions of the *Vegfr3* gene via Ets binding to DNA.

## Discussion

Previously, Wei et al. demonstrated that genetically engineered mice lacking both Ets1 and Ets2 die at mid-gestation and exhibit defects in vascular branching of blood vessels and impaired blood endothelial cell (BEC) survival [38]. However, lymphangiogenesis in those mice was not studied. On the other hand, Yoshimatsu et al. reported that Ets family proteins can physically interact with Prox1 and co-localize on the *VEGFR3* promoter in human LECs [13]. They also demonstrated that adenoviral overexpression of Ets1/2 proteins in human LECs led to the up-regulation of *VEGFR3* gene expression [13], suggesting that Ets family proteins, together with Prox1, may up-regulate *VEGFR3* gene expression in LECs. However, the role of endogenous Ets1/2 proteins and intracellular signaling upstream of Ets in LECs remained unknown.

**Figure 7:**
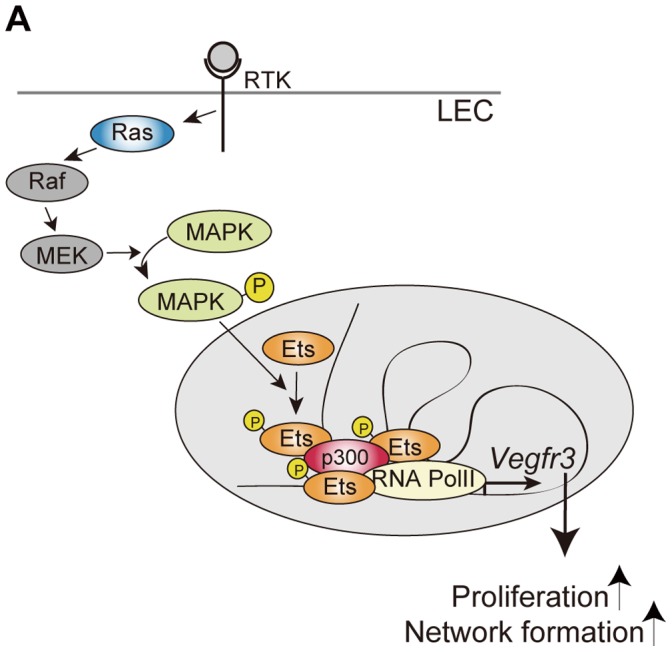
Schematic representation of Ets-mediated transcriptional regulation of *Vegfr3*. The Ras/MAPK/Ets pathway involved in transcriptional regulation of the *Vegfr3* gene in LECs. Ras/MAPK signal-activated Ets proteins, together with p300, regulate *Vegfr3* gene expression and lead to changes in cellular proliferation and morphogenesis.

In the present study, we provide evidence that endogenous Ets1 and Ets2 regulate transcription of the *Vegfr3* gene by binding to its non-coding regulatory regions and mediating p300 recruitment to the gene. Based on our findings described above, we propose a Ras-mediated signaling mechanism that modulates *Vegfr3* gene expression and LEC characteristics, as illustrated in Fig. 7. The mechanism that we propose in the present study is: 1) Ras signaling activates Ets proteins via MAPK-induced phosphorylation (Fig. 1); 2) activated Ets proteins bind to non-coding regulatory elements within the *Vegfr3* gene that harbor GGAA/T motifs (Fig. 4 and Fig. 5); 3) DNA-bound Ets proteins bind p300 and form an Ets/p300 complex on the *Vegfr3* gene (Fig. 6); 4) p300 acetylates H3 histones in the chromatin of the *Vegfr3* gene and activates transcription from a core promoter (Fig. 5C); 5) altered VEGFR-3 expression modulates LEC phenotypes such as cell proliferation and network formation (Fig. 2 and Fig. 3).

We and others found previously that intracellular signaling through VEGFR-3 was altered in Ras-knockdown, Ras-overexpressing (e.g., Ras-hyperactive), or Rasa1-deficient LECs, indicating that Ras signaling integrates into downstream signaling of VEGFR-3 in conventional receptor tyrosine kinase (RTK)/Ras/MAPK signaling [26], [39]. Nevertheless, accumulating evidence has implied that LEC participation in development, inflammation, tissue repair and tumorigenesis may be modulated or fine-tuned by VEGFR-3 expression levels [10], [11], [12], [13], [14], as well as by intracellular signaling through VEGFR-3. As demonstrated here, Ras/MAPK signaling directs *Vegfr3* gene expression by regulating Ets-mediated transcription activity. To understand the mechanism by which VEGFR-3 expression levels are regulated during developmental, physiological and pathological lymphangiogenesis, it will be important to identify the upstream signals that positively or negatively regulate Ras/MAPK/Ets signaling involved in *Vegfr3* gene expression events.

As Ets knockdown downregulates *Vegfr3* gene expression, VEGFR-3 protein levels appear to be reduced correspondingly (Fig. 1). However, the expression level of VEGFR-3 protein was not reduced as much as that of *Vegfr3* mRNA when examined at 72 hr after transfection with Ets siRNA (Fig. 1). Although real-time RT-PCR and western blotting are semi-quantitative methods and thus the exact quantities of protein and mRNA remain to be precisely compared, this result suggests that VEGFR-3 protein turnover might be slower than *Vegfr3* mRNA turnover, and that remaining VEGFR-3 protein may attenuate the effect of Ets-knockdown on mLECs. Constitutive expression of shRNA specific to Ets genes or deletion of Ets genes should be employed in future studies to circumvent this issue.

Although our findings strongly suggest that Ets1/2 proteins are responsible for *Vegfr3*/*VEGFR3* gene expression and LEC phenotypes in both mice and humans, we also found a discrepancy between mLECs and hLECs that remains unsolved. hLECs but not mLECs were affected severely by Ets1 knockdown, whereas mLECs but not hLECs were sensitive to Ets2 knockdown (Fig. 2, Fig. 3 and Fig. 4). This discrepancy might result from different roles of the *Ets*/*ETS* genes in the two species; however, two additional differences between mLECs and hLECs must be addressed. As described in the Materials and Methods, we used immortalized mouse LECs that express a weakly-acting, thermo-labile mutant of the SV40 large T antigen (tsA58 T antigen). In this study, we cultured these cells at 33°C, the permissive temperature for the mutant protein, since these cells proliferate uniformly at 33°C, but not at 37°C, without losing the characteristics of LECs. The SV40 T antigen is known to suppress p53- and Rb-dependent cellular responses, such as cell cycle arrest and apoptosis. Although the mechanisms by which p53 and Rb-dependent intracellular responses crosstalk with the Ras/MAPK/Ets pathway are unknown, it is possible that the SV40 T antigen might partially rescue Ets1-knockdown mLECs from cell growth arrest (Fig. 2) and suppression of transcriptional regulation (Fig. 4). Alternatively, the mLECs and hLECs used in this study were obtained from the mesentery and the skin, respectively. Through the phenotypic characterization of knockout mice, previous studies have reported tissue/organ-specific lymphangiogenic disorders, implying heterogeneity of LEC characteristics in different tissues/organs. The discrepancy regarding the role of Ets1/ETS1 versus Ets2/ETS2 in mLECs and hLECs remains to be explored.

As described above, we identified non-coding regulatory regions within *Vegfr3* that are evolutionarily conserved between species, harbor Ets-binding motifs, and have enhancer activities as shown by luciferase reporter assay. ChIP experiments suggested that Ets and p300 associate with corresponding regions in the intact chromatin of LECs. In *Vegfr3*-EGFP knock-in mice harboring mutations in the Ets-binding motifs of the 5′ regulatory region, neither EGFP expression levels nor pattern were affected significantly compared to wild-type *Vegfr3*-EGFP knock-in mice (Ichise, T., unpublished observation). In transgenesis, we and others have shown that the *Vegfr3* promoter that includes the 5′ regulatory region is sufficient to drive LEC-specific transcription *in vivo*. However, *Vegfr3* promoter-driven reporter expression was not only weaker than expression from a *Vegfr3* reporter knock-in allele, but was also sensitive to “position effects”, which are the effects of epigenetic modifications in genomic regions surrounding integrated transgenes that alter expression patterns and transcription levels of those transgenes (Shiozawa, S. and Ichise, H., unpublished observation). These results imply that regulatory regions other than the 5′ untranscribed region might be required to maintain proper expression levels and overcome negative regulation by epigenetic modification. Multiple regulatory regions might work cooperatively and/or redundantly in transcriptional regulation of the *Vegfr3* gene. Deletions or nucleotide mutations of these regions on the endogenous allele or a BAC transgene can be used to address this question.

Based on results obtained in ChIP assays, Pan et al. and Yoshimatsu et al. suggested that Prox1 may bind to the region between HR1 and HR2 in the human *VEGFR3* gene in Prox1-overexpressing EA.hy926 cells and human LECs, respectively [13], [23]. Although the sequences in this region are not evolutionally conserved, this region in the mouse *Vegfr3* gene contains potential Prox1-binding sites similar to putative Prox1-binding consensus sequences (data not shown). Prox1 may participate in *Vegfr3* gene expression by binding to this region and associating with Ets proteins that are bound to regulatory elements within the *Vegfr3* gene. VEGFR-3 is also expressed transiently in embryonic BECs [6] and a subset of BECs during tip-stalk cell specification in angiogenesis [40], neither of which express Prox1, indicating that the *Vegfr3* gene in these ECs is under the control of Prox1-independent transcriptional regulation. The prominent vascular phenotypes observed in Ras knockout mice and Ras-overexpressing mice in an EC lineage are restricted to lymphatic vessels, as described previously [26]. Thus, the Ras/MAPK/Ets pathway responsible for regulating *Vegfr3* transcriptional activity and LEC phenotypes may require LEC-specific co-factor(s) and/or epigenetic programming of chromatin in LECs. It remains to be elucidated whether the Ras/MAPK/Ets pathway is involved in *Vegfr3* gene expression in BECs as well as LECs, and whether a functional relationship exists between the Ras/MAPK/Ets pathway and Prox1 activity in LECs.

In this study, the region designated HR10, which is the evolutionarily conserved region between exon 11 and 12, harbors a FOX:ETS motif followed by a Tbx1-binding site (Fig. S1 and Fig. S2) [25], [34]. Although a FOX:ETS motif has been suggested to play an important role in the specification and maintenance of EC-specific gene expression by the Ets family transcription factor, Etv2, the significance of the presence of this motif in the *Vegfr3* gene had not been studied [34]. However, Tbx1-dependent enhancer activity of this region in ECs has been reported previously [25]. In this study, we found that HR10 has an enhancer activity and binds to both Ets and p300 proteins in LECs, strongly suggesting that the region may be important for *Vegfr3* gene expression. It remains to be elucidated whether crosstalk occurs between Ets and Fox family- and Tbx1-dependent transcriptional networks via the HR10 region.

In addition to the role of Ras/MAPK/Ets in LECs, we also discovered a role for p300 and a functional difference between two types of HATs, p300 and CBP, in *Vegfr3* gene expression events in LECs. Histone H3 acetylation has been shown to correlate positively with the transcriptional activation of genes. Even though it remains uncertain whether histone acetylation plays an active role in chromatin remodeling for transcription, it is probable that Ets-induced p300 recruitment is important for the transcriptional activation of the *Vegfr3* gene. On the other hand, CBP does not appear to be involved in the maintenance of *Vegfr3* gene expression. Both p300 and CBP are reported to be co-activators of Ets in transcriptional activation and to bind phosphorylated Ets [36], [37]. It remains unknown why CBP knockdown did not affect *Vegfr3* gene expression. Although we cannot compare the expression levels of p300 with those of CBP due to a lack of antibodies able to simultaneously detect both proteins, one explanation may be that p300 might be expressed dominantly in LECs. Alternatively, other binding partners or protein modifications might modulate the interaction between Ets and p300/CBP, leading to functional differences between the Ets/p300 and Ets/CBP complexes. Intriguingly, a previous study reported that edema and hemorrhaging were observed in acetyltransferase activity (AT)-deficient mice heterozygous for p300 at E15, and that those defects were resolved by E18.5 [41]. It is speculated that edema and hemorrhage might be caused by lymphatic vessel malformation caused by the functional deficiency of p300 in LECs. In the future, it will be important to investigate the roles of p300 and CBP in Ets-mediated transcriptional regulation of *Vegfr3* in LECs *in vivo*.

## Materials and Methods

### Cell Culture

Immortalized mouse mesenteric LECs, hereafter referred to as mLECs, were described previously [26]. Briefly, according to the procedure described previously [42], SV40 tsA58T antigen-expressing LECs were obtained from mesenteries of 10–20 day-old CAG-βgeo-tsA58T Ag; EC-specific Cre double transgenic mice. H-Ras-overexpressing LECs were obtained from CAG-βgeo-tsA58T Ag; EC-specific Cre; CGH triple transgenic mice. Lyve-1-positive ECs were isolated using magnetic immunosorting and maintained at 33°C (a permissive temperature for the thermo-labile tsA58T antigen). Pan-EC- and LEC-specific marker expression (VEGFR-2, CD31 and VE-cadherin for pan-EC-specific markers; VEGFR-3, Lyve1, Prox1 and Podoplanin for LEC-specific markers) was verified by western blotting or immunocytochemistry. Cells were cultured in EGM-2MV medium (Lonza, Basel, Switzerland) on gelatinized culture dishes at 33°C with 5% CO_2_ in a humidified incubator. Human dermal microvascular LECs (hLECs) from pooled donors (Lonza) were cultured on gelatinized culture dishes using an EGM-2 MV bullet kit (Lonza) according to the manufacturer’s protocol. The Cell Proliferation Reagent WST-1 (Roche Diagnostics K. K. Tokyo, Japan) and a CycLex Cellular BrdU ELISA Kit (CycLex Co., Ltd., Nagano, Japan) were used according to the manufacturer’s instructions.

### Mice and Ethics Statement

C57BL/6J mice were purchased from CLEA Japan (Tokyo, Japan) and incrossed to obtain newborn mice. All mice were housed under pathogen-free conditions in the experimental animal facility at the University of Tokyo. All procedures for isolating mouse LECs in our previous studies [26], [42] and obtaining mouse tissue samples in this study were approved by the Animal Care and Use Committee of the University of Tokyo and conducted in accordance with their guidelines (approval nos. PA11-94 and PA11-95).

### Knockdown Experiments

Knockdown experiments were performed using Stealth RNAi siRNA duplexes, the corresponding Stealth RNAi Negative Control Duplexes and Lipofectamine RNAiMAX transfection reagent (Invitrogen/Life Technologies, Carlsbad, CA, USA) according to the manufacturer’s instructions. Prior to knockdown experiments, transfection was optimized by monitoring transfection efficiency of the BLOCK-iT Alexa Fluor Red Fluorescent Control (Invitrogen/Life Technologies). For mLECs, Stealth RNAi siRNA duplexes (Invitrogen/Life Technologies) for *Hras*, *Nras*, *Kras*, *Flt4*, *Ets1* (MSS280297, MSS280295), *Ets2* (MSS280301, MSS280302), *Ep300* (MSS220767, MSS220768) and *Crebbp* (MSS273799, MSS273800) were used. The sense-strand sequences of the siRNA duplexes for *Hras*, *Nras* and *Kras* were described previously [26]. The sense-strand sequences of the two siRNA duplexes for *Vegfr3* were 5′-UACAGGAGCGGUACAGAGUUCAAGG-3′ (si-1) and 5′-AUAUGUAUUCCUUAUGAGAACCCUG-3′ (si-2). Cells were transfected with 20 nM siRNA duplexes for 24 hr and then cultured in EGM-2MV medium for 48 hr (totally 72 hr after transfection). Cells cultured for 72 h after transfection were used for analyses.

For hLECs, Stealth RNAi siRNA duplexes for ETS1 (HSS103402, HSS103403) were used. Cells were transfected with 60 nM siRNA duplexes for 24 hr. Cells cultured for 48 h after transfection were used for analyses.

### Western Blot Analysis

Cell lysates (20 µg) were resolved by SDS-PAGE, and blotted by semi-dry transfer onto a PVDF (Millipore, Billerica, MA, USA) or nitrocellulose (Bio-Rad Laboratories, Hercules, CA, USA) membrane. For Fig. 6A, nuclear extract lysates (10 µg) were prepared using NE-PER Nuclear and Cytoplasmic Extraction Reagents (Pierce Biotechnology/Thermo Scientific, IL, USA) and used for the detection of p300 and CBP. Western blot analysis was performed using the following primary antibodies: mouse anti-Pan-Ras (Calbiochem/Merck, Darmstadt, Germany), rat anti-VEGFR-3 (eBioscience, San Diego, CA, USA), rabbit anti-Ets1, rabbit anti-Ets2, rabbit anti-p300, and rabbit anti-CBP (Santa Cruz Biotechnology, Santa Cruz, CA, USA), mouse anti-Gapdh (Millipore) or rabbit anti-phosphorylated Ets1 (Biosource/Invitrogen). The secondary antibodies used were swine anti-goat IgG (HRP), goat anti-rabbit IgG (HRP), anti-hamster IgG (HRP) and goat anti-rat IgG (HRP) (Biosource/Invitrogen). Signals were detected using ECL (GE Healthcare Bio-Sciences, Piscataway, NJ, USA) and X-ray film (Fujifilm, Tokyo, Japan). For Western blot analysis, three independent samples from the same experiment (triplicates) were separated on the same gel and transferred onto the same membrane. Western blot results shown in the figures are representative of triplicate experiments. Western blot images were analyzed using NIH Image J software, and quantitative analyses are represented graphically. Each western blot/quantification analysis was performed by at least two independent experiments to confirm the reproducibility of the experiment. For Fig. 1C and Fig. 2A, the band intensities of western blots were first normalized to that of Gapdh, and then normalized to the expression levels of LECs transfected with control siRNA.

### Quantitative Real-time RT-PCR

Total RNA from LECs was obtained using TRIzol reagent (Invitrogen), treated with DNase I and purified. cDNA corresponding to 20 ng of total RNA was used for each reaction. Quantitative real-time RT-PCR was performed with the StepOne Real-time PCR system using a High-Capacity cDNA Reverse Transcription kit, TaqMan Gene Expression Master Mix, TaqMan Gene Expression Assays (*Flt4* (*Vegfr3*), Mm00433337_m1; *Ets1*, Mm01175819_m1; *Ets2*, Mm00468977_m1; *Ep300*, Mm00625535_m1; *Crebbp*, Mm01342452_m1; *Gapdh*, Mm99999915_g1; *Hprt*, Mm00446968_m1; *Tbp*, Mm00446973_m1; *Kdr* (*Vegfr2*), Mm01222419_m1; *Pecam1*,Mm01242584_m1; *Cdh5* (*VEcadherin*), Mm00486938_m1; *FLT4*, Hs01047677_m1; *ETS1*, Hs00428293_m1; and TaqMan Endogenous control 18S rRNA (Applied Biosystems/Life Technologies, Carlsbad, CA, USA) according to the manufacturer’s protocols. Triplicates were run and analyzed using the comparative Ct method, according to manufacturer’s protocol.

Briefly, for the comparative Ct method, TaqMan Endogenous Control 18S rRNA reagent was used, and the gene-of-interest (GOI) Ct and 18s rRNA Ct values were obtained simultaneously by multiplex PCR. The quantity of target rRNA was normalized to the endogenous control (18S rRNA) and is presented relative to a reference sample (control siRNA-transfected cells). The ΔCt value was determined by subtracting the average 18S rRNA Ct value from the average GOI Ct value, and the ΔΔCt value was then determined by subtracting the ΔCt of a reference sample from the ΔCt of a test sample. The relative quantity, identified as the “relative mRNA expression” in the figures, was determined by 2^–ΔΔCt^. The means of the relative quantity of control siRNA-transfected cells were set to 1. In Fig. S1, the relative *Vegfr3* mRNA levels were further normalized to the relative mRNA levels of the housekeeping genes, *Gapdh*, *Hprt* or *Tbp*.

### Luciferase Reporter Assays

A DNA fragment 3,035-bp (Kpn I/Not I) in length harboring the mouse *Vegfr3* promoter region derived from the RPCI23-118J11 BAC DNA (ResGen/Invitrogen) was subcloned into pGL4.10 (Promega, Madison, WI, USA) and used to generate deleted versions of the reporter constructs [817 bp (Avr II/Not I), 451 bp (Bgl II/Not I) and 210 bp (BsiWI/Not I) and delta HR2 reporter constructs] by restriction enzyme digestion, modification of restriction enzyme recognition sites and re-ligation. Tandem G-to-A mutations were introduced into two putative Ets-binding sites (GGAA; the resulting mutated sequence is CCAA) using the PrimeSTAR Mutagenesis Basal Kit (TaKaRa Bio, Shiga, Japan), according to the manufacturer’s instructions. The mutated fragments were sequence-verified and recloned into the original, non-mutated versions of the reporter constructs to avoid potential unwanted mutations in the plasmids. Mouse *Vegfr3* intronic regions with highly conserved sequences were identified using the UCSC Genome Browser. Results for multiple alignment and conservation between species are shown in Fig. S2 and Fig. S3. HR regions were defined as non-repetitive regions evolutionarily conserved among more than three species. The HR regions were PCR-amplified using mouse genomic DNA as a template, and subcloned into the *Vegfr3* AvrII/NotI luciferase constructs described above. The primers used for cloning are listed in Table S1 (*Vegfr3* HR3, HR5, HR6, HR10). For luciferase assays, immortalized mouse LECs were seeded in 6-well plates at a density of 2.8×10^5^ cells per well. Equal amounts (2 µg) of the different *Vegfr3* luciferase constructs and 20 ng of the Renilla luciferase expression vector pGL4.74 (Promega) were transfected using Fugene HD Transfection Reagent (Roche Diagnostics). After 24 hr, the cells were lysed and luciferase activity was measured using a Dual Luciferase Assay kit (Promega) and a SIRIUS luminometer (Berthold Japan, Tokyo, Japan). All experiments were performed in triplicate.

### ChIP Assay

Immortalized mouse LECs (0.6×10^6^) were seeded in EGM-2MV medium on three 10-cm^2^ gelatinized culture dishes at 33°C. The next day, the cells were transfected with siRNA for 24 hr and then cultured in EGM-2MV medium for 48 hr. Small intestines of newborn mice were processed according to Farnham Lab ChIPs Protocol for Tissues (2006 Revision; http://farnham.genomecenter.ucdavis.edu/protocols/tissues.html) with minor modifications. The cells and tissue samples were lysed and sonicated using a BIORUPTOR UCD-250 (Cosmo Bio, Tokyo, Japan) to generate the chromatin preparation, and ChIP assays were performed using a ChIP assay kit (Millipore) according to the manufacturer’s instructions. The average size of the sonicated input DNA was approximately 500 bp. Lysates were immunoprecipitated with anti-acetyl histone H3 (Upstate/Millipore), anti-Ets1 (Santa Cruz Biotechnology), anti-p300 (Santa Cruz Biotechnology) or normal Rabbit IgG (Upstate/Millipore). Three (for acetyl histone H3, Ets1 and Rabbit IgG) or six (for p300) replicates of each ChIP experiment from independent cell cultures were performed. A fraction (1%) of the sonicated chromatin was set aside before the antibody affinity manipulations as ‘input’ DNA. The resulting enriched and input DNA was purified following cross-link reversal, and then analyzed by qPCR (Power SYBR, Applied Biosystems) using primers specific for the *Vegfr3* gene and the *Gapdh* promoter. The primers used for the ChIP assays are listed in the Table S1 (*Vegfr3* HR1, HR3, HR5, HR6, HR10 and *Gapdh*). Percent input was calculated by the equation: 100×2?(Ct_adjusted Input_ – Ct_Enriched_). Input DNA Ct was adjusted from 1% to 100% by subtracting 6.644 Cts or Log_2_ 100.

### LEC Network Formation on Matrigel

Twelve-well Matrigel (BD Pharmingen, Franklin Lakes, NJ, USA)-coated dishes (9.5 mg/ml; 200 µl per well) were used to analyze network formation by LECs. mLECs and hLECs were cultured and incubated at 33°C and 37°C, respectively. mLECs were transfected with siRNA for 24 hr and cultured in EGM-2MV medium for 24 hr, then 1 µg/ml DiI (Molecular Probes/Invitrogen) was added to the medium. hLECs were transfected with siRNA for 24 hr, then 1 µg/ml DiI was added to the medium. The cells were incubated for 30 min, then washed twice in PBS and trypsinized. The DiI-labeled mLECs and hLECs were counted, seeded on Matrigel-coated dishes at a cell concentration of 4×10^4^ cells and 3×10^4^ cells per well, respectively, and cultured in EGM-2MV medium (Ronza) for 24 hr. The 1-cm^2^ areas covered by DiI-labeled cellular structures were measured using a BioRevo BZ-9000 imaging system (Keyence, Osaka, Japan). Three independent wells were used for each analysis.

### Statistics

Comparisons in this study were made using the two-tailed paired Student’s t-test (α = 0.05). For quantitative real-time RT-PCR, statistical analyses were performed at the ΔCt stage.

## Supporting Information

Figure S1 ***Vegfr3*** **gene expression is dependent on Ets1 and Ets2 in mLECs.** A. Real-time RT-PCR assay for mRNAs in mLECs transfected with control, Ets1 and Ets2 siRNAs. si-1 and -2 represent two individual siRNAs. Ets1/2 si represents transfection with mixed siRNAs for Ets1 and Ets2. Error bars represent the S.D.; n = 3. *p<0.05, ***p<0.005, ****p<0.001 (vs. mLECs transfected with control siRNA; see Table S4 and Table S5). B. Real-time RT-PCR assay for *Vegfr3* mRNA in mLECs transfected with control, Ets1 and Ets2 siRNAs. *Vegfr3* mRNA levels are normalized to *Gapdh*, *Hprt*, or *Tbp* mRNA levels. Error bars represent the S.D.; n = 3. *p<0.05, ***p<0.005, ****p<0.001 (vs. mLECs transfected with control siRNA; see Table S2, Table S3, Table S4 and Table S5).
(TIF)

Figure S2 **Low-power field representation of the evolutionarily conserved regions HR1-12 within the mouse** ***Vegfr3*** **gene.** Using the DNA sequences of fragments that were used in luciferase assays and PCR-amplified in ChIP assays in this study with those of previously reported regulatory regions (HR1, HR2, FOX:ETS and the Tbx1-binding site), a BLAT search was performed. The results are shown using the UCSC Genome Browser.
(JPG)

Figure S3 **High-power field representation of genomic fragments used in luciferase assays and PCR-amplified in ChIP assays.** The bars below each alignment indicate evolutionarily conserved GGAA/T motifs. Mutated sites in Fig. 4C (mut1 and mut2) are also indicated here.
(JPG)

Figure S4 **Confirmation of the effects of p300- and CBP-knockdown on gene expression in mLECs.** Real-time RT-PCR assay for *Vegfr3*, *p300*, *CBP, Ets1*, and *Ets2* mRNAs in mLECs transfected with control, p300, and CBP siRNAs (si-2). Error bars represent the S.D.; n = 3. *p<0.05, ****p<0.001 (vs. LECs transfected with control siRNA; see Table S8).
(TIF)

Table S1 **Primer sequences for ChIP assays.**
(XLSX)

Table S2 **ΔCt values and P-values for statistical analysis at the ΔCt stage in** **Figure 1B** **(si-1).**
(XLSX)

Table S3 **ΔCt values and P-values for statistical analysis at the ΔCt stage in** **Figure 1B** **(si-2).**
(XLSX)

Table S4 **ΔCt values and P-values for statistical analysis at the ΔCt stage in Figure S1 (si-1).**
(XLSX)

Table S5 **ΔCt values and P-values for statistical analysis at the ΔCt stage in Figure S1 (si-2).**
(XLSX)

Table S6 **ΔCt values and P-values for statistical analysis at the ΔCt stage in** **Figure 3A** **.**
(XLSX)

Table S7 **ΔCt values and P-values for statistical analysis at the ΔCt stage in** **Figure 6B** **.**
(XLSX)

Table S8 **ΔCt values and P-values for statistical analysis at the ΔCt stage in Figure S4.**
(XLSX)
