# Supplementary figures and images for: Ras/MAPK Signaling Modulates VEGFR-3 Expression through Ets-Mediated p300 Recruitment and Histone Acetylation on the Vegfr3 Gene in Lymphatic Endothelial Cells
Source: PLoS One. 2012 Dec 17;7(12):e51639. doi: 10.1371/journal.pone.0051639 (PMC3524184; doi:10.1371/journal.pone.0051639)

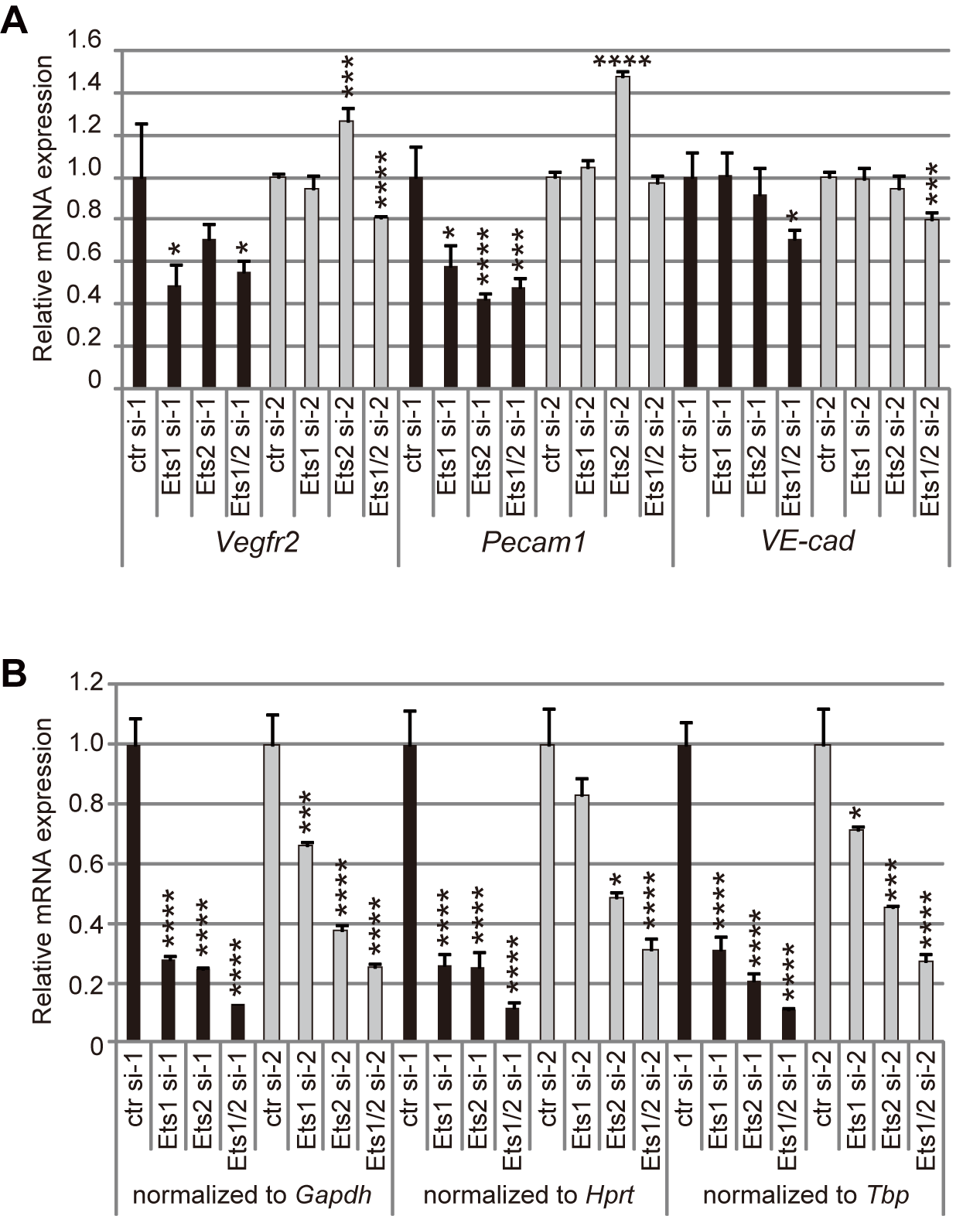

Supplement: Figure S1 — Vegfr3 gene expression is dependent on Ets1 and Ets2 in mLECs. A. Real-time RT-PCR assay for mRNAs in mLECs transfected with control, Ets1 and Ets2 siRNAs. si-1 and -2 represent two individual siRNAs. Ets1/2 si represents transfection with mixed siRNAs for Ets1 and Ets2. Error bars represent the S.D.; n = 3. *p<0.05, ***p<0.005, ****p<0.001 (vs. mLECs transfected with control siRNA; see Table S4 and Table S5). B. Real-time RT-PCR assay for Vegfr3 mRNA in mLECs transfected with control, Ets1 and Ets2 siRNAs. Vegfr3 mRNA levels are normalized to Gapdh, Hprt, or Tbp mRNA levels. Error bars represent the S.D.; n = 3. *p<0.05, ***p<0.005, ****p<0.001 (vs. mLECs transfected with control siRNA; see Table S2, Table S3, Table S4 and Table S5). (TIF) [file pone.0051639.s001.tif]

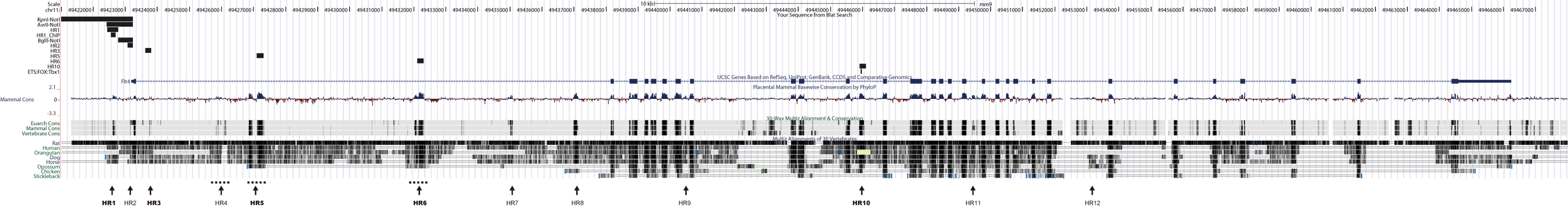

Supplement: Figure S2 — Low-power field representation of the evolutionarily conserved regions HR1-12 within the mouse Vegfr3 gene. Using the DNA sequences of fragments that were used in luciferase assays and PCR-amplified in ChIP assays in this study with those of previously reported regulatory regions (HR1, HR2, FOX:ETS and the Tbx1-binding site), a BLAT search was performed. The results are shown using the UCSC Genome Browser. (JPG) [file pone.0051639.s002.jpg]

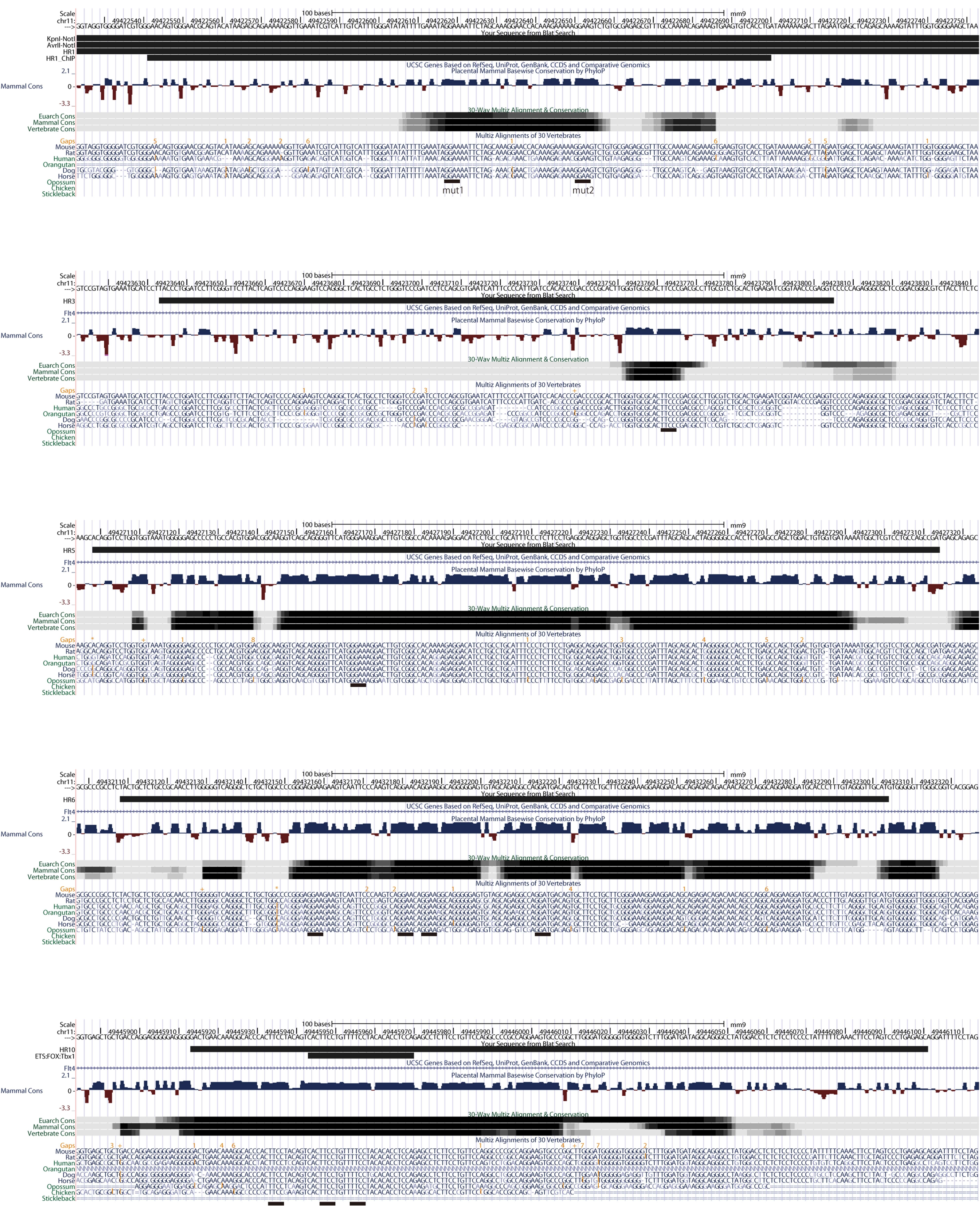

Supplement: Figure S3 — High-power field representation of genomic fragments used in luciferase assays and PCR-amplified in ChIP assays. The bars below each alignment indicate evolutionarily conserved GGAA/T motifs. Mutated sites in Fig. 4C (mut1 and mut2) are also indicated here. (JPG) [file pone.0051639.s003.jpg]

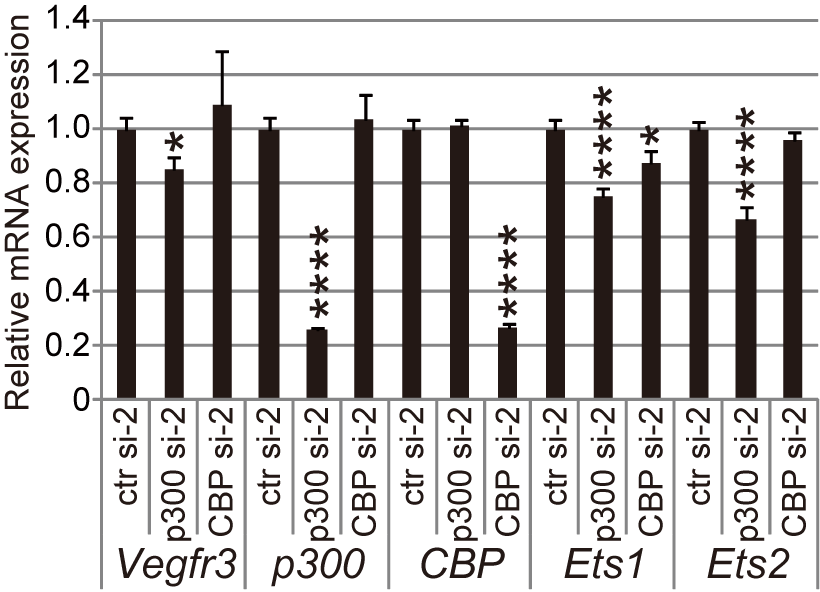

Supplement: Figure S4 — Confirmation of the effects of p300- and CBP-knockdown on gene expression in mLECs. Real-time RT-PCR assay for Vegfr3, p300, CBP, Ets1, and Ets2 mRNAs in mLECs transfected with control, p300, and CBP siRNAs (si-2). Error bars represent the S.D.; n = 3. *p<0.05, ****p<0.001 (vs. LECs transfected with control siRNA; see Table S8). (TIF) [file pone.0051639.s004.tif]
